# Plant Food Residues as a Source of Nutraceuticals and Functional Foods

**DOI:** 10.3390/foods5040088

**Published:** 2016-12-10

**Authors:** Theodoros Varzakas, George Zakynthinos, Francis Verpoort

**Affiliations:** 1 TEI Peloponnese, Department of Food Technology, Kalamata 24100, Greece; gzakyn@yahoo.gr; 2 Department of Bioscience Bioengineering, Global Campus Songdo, Ghent University, 119 Songdomunhwa-Ro, Yeonsu-Gu, Incheon 406-840, Korea; Francis.Verpoort@ghent.ac.kr; 3 Laboratory of Organometallics, Catalysis and Ordered Materials, State Key Laboratory of Advanced Technology for Materials Synthesis and Processing, Wuhan University of Technology, Wuhan 430070, China; 4 National Research Tomsk Polytechnic University, Lenin Avenue 30, Tomsk 634050, Russia

**Keywords:** plant, vegetable, food residues, nutraceuticals, functional foods

## Abstract

This chapter describes the use of different plant and vegetable food residues as nutraceuticals and functional foods. Different nutraceuticals are mentioned and explained. Their uses are well addressed along with their disease management and their action as nutraceutical delivery vehicles.

## 1. Introduction

Fruit and vegetable wastes (FVW) are produced in large quantities in markets, and constitute a big problem in municipal landfills due to their high biodegradability.

Fruit juices and derived products such as nectars and drinks have experienced growing popularity within the last years. Grapes and apples are the most important fruits in the temperate zone, while oranges, pineapples, bananas, watermelons and mangos are the predominant fruits of tropical and subtropical areas [1].

Fruits from the temperate zone are usually characterized by a large edible portion and moderate amounts of waste material such as peels, seeds and stones. In contrast, considerably higher ratios of by-products arise from tropical and subtropical fruit processing. Due to increasing production, disposal represents a growing problem since the plant material is usually prone to microbial spoilage, thus limiting further exploitation. On the other hand, costs of drying, storage and shipment of by-products are economically limiting factors. Therefore, agro-industrial waste is often utilized as feed or as fertilizer.

Consumers are aware of diet-related health problems and need to increase consumption of fruits and vegetables, however, this is not so feasible in our everyday life. Hence dietary supplements may offer an alternative to the access of compounds such as carotenoids, polyphenolics, tocopherols, and vitamin C.

At present, agricultural by-products are mainly used as combustion feedstock for biofuels [2,3]. The most important biomasses are the residues from woodworking (wood shavings, sawdust) or forest activities, the wastes from farms and agro-business, the organic fraction of municipal solid wastes, and the plants deliberately grown for energetic purposes. In Italy, the most utilized biomasses for burning in power plants are chipped wood, and, to a minor extent, rice-husk and olive residues [2,3].

Vegetables and some fruits yield between 25% and 30% of nonedible products [4,5]. The by-products of fruits and vegetables are made up of skins and seeds of different shapes and sizes that normally have no further usage and are commonly wasted or discarded [4].

Preliminary studies conducted by Ayala-Zavala et al. [6] demonstrated that several kinds of fresh-cut fruits produced variable amounts of by-products to the extent even exceeding the quantity of end produce. The processed fruits were apples (*Malus domestica* cv. Golden Delicious), mandarins (*Citrus reticulata*), papayas (*Carica papaya* cv. Maradol), pineapples (*Ananas comosus* cv. Premium cayenne), and mangos (*Mangifera indica* cv. Kent). Sliced apples produced 10.91% of pulp and seed (core) by-products and 89.09% of the final products. Peeled mandarins produced 16.05% of peels and 83.95% of final products. Diced papayas produced 6.51% of seeds, 8.47% of peels, 32.06% of unusable pulp (due to the lack of shape uniformity in a cube), and 52.96% of final products.

Pineapples produced 9.12% of core, 13.48% of peels, 14.49% of pulp, 14.87% of top, and 48.04% of finished products. Mangos produced 13.5% of seeds, 11% of peels, 17.94% unusable pulp, and 57.56% of final products.

It has been reported that the total phenolic compounds of seeds of fruits, such as mangos, longans, avocados, and jackfruits, were higher than that of the edible product, and that a valuable source of phytochemicals could be provided by the by-products [7]. The peels and seeds of tomatoes are richer sources of phenolic compounds compared to their pulp.

## 2. Use of Plant Food Residues as Functional Foods—Phytochemicals Presence

Products isolated or purified from food but that are generally sold in medicinal forms not usually associated with food, such as capsules, are referred to as nutraceuticals. Nutraceuticals are shown to exert a physiological benefit or provide protection against chronic disease. A working definition of nutraceutical from a Science forum states: “a diet supplement that delivers a concentrated form of a biologically active component of food in a non-food matrix to enhance health” [8]. According to the forum functional foods “are consumed as part of a normal diet and deliver one or more active ingredients (that have physiologic effects and may enhance health) within the food matrix.

According to the claims made by the European regulation on nutrition and health on foods (EC No. 1924/2006), a list of authorized claims has to be published for all member states, and nutrient profiles also should be established for foods containing health claims [9].

In the United States, a product is classified and regulated as food, food supplement, diet constituent, or nutritional supplement on the basis of its components and the tag or nutritional label with which the product is marketed (http://www.fda.gov/Food/DietarySupplements/QADietarySupplements/default.htm#top; http://www.nap.edu/ openbook.php?record_id=10882).

Under the Canadian law, a “nutraceutical” or “functional food” has no legal distinction and both of these can either be marketed as a food or as a drug. Drug and food products must comply with all the quality and safety requirements of the Food and Drug Regulations. In addition to quality and safety requirements, drugs may be approved for sale if they meet the regulatory requirements for efficacy. The law describes nutraceutical as a product derived or refined from nutrients that is marketed in medicinal form but not typically connected with food and is validated to provide a functional benefit or a shield against protracted illness. Functional food, according to this law, is visually comparable with or may be a traditional food consumed in the everyday diet and is revealed to provide bodily health in addition to basic nutritional functions. These foods may also facilitate the reduction of the threat of chronic ailment [10].

This confusion in the literature has been clarified by González-Sarrías et al. [11] by differentiating functional foods, nutraceuticals, medical foods, and botanicals.

Functional foods are those that provide a scientifically proven specific health benefit (health claim) beyond their nutritional format. These include processed foods or foods with health-promoting additives such as the addition of iodine to table salt or vitamin D to milk.

Dietary (or food) supplements are products that are consumed to complement dietary (or food) supplements. They are not intended to treat, diagnose, mitigate, prevent, or cure diseases. Besides other substances, they include fatty acids, vitamins, minerals, amino acids, or fiber. It is also believed that more than 50,000 dietary supplements are available, mostly multivitamins. They are consumed in a pharmaceutical format that is, pill, tablet, powder, but not in the form of beverages (soups, juices, etc.) or conventional foods.

Antioxidant plant pigments, primarily carotenoids and flavonoids, are also called phytochemicals. Phytochemicals is a broad name that can be used for a variety of compounds produced by plants. Allyl sulfides, anthocyanidins, catechins, carotenoids, flavonoids, flavones, isoflavones, isothiocyanates, phytonutrients, and polyphenols are amongst these compounds for phytochemicals. It is believed by researchers that there are some 4000 phytochemicals and they all are found in vegetables, fruit, beans, and grains [11].

A balanced intake of vegetables, fruit, beans, and grains can provide a variety of beneficial compounds. New studies have demonstrated that there are multiple effects of vegetables and fruits e.g., low concentration in fat, salt, and sugar or a good source of dietary fiber. They contain many vitamins and minerals that are good for health, such as vitamin A (beta-carotene), C and E, magnesium, phosphorous, folic acid, and zinc.

Flavonoids are a group of plant metabolites that belong to the most common group of polyphenolics in the human diet. They are subdivided into several other groups including flavone, flavonol, flavanone, and isoflavones.

Recent research suggests that, in humans, these plant polyphenols provide important health benefits related to metabolic syndrome, cancer, brain health, and the immune system.

The citrus flavonoids, naringin, and naringenin, were found to significantly lower the expression levels of vascular cell adhesion molecule-1 (VCAM-1) and monocyte chemotactic protein-1 (MCP-1), with potential applications in the prevention of atherosclerosis [12]. Bioflavonoids such as hesperidin (from orange peel), naringin (grapefruit peel), or rutin can normalize capillary permeability and vascular brittleness, and hence can be called vitamin P factors [13].

The flavanols (e.g., catechins, proanthocyanidins) comprising the phenolic compounds of wine, have been the focus of a number of studies due to the beneficial effects exerted by a moderate consumption of wine [14]. These compounds have their origin in grape, and only a part of them is transferred to the must. Their extractability mainly depends on the technological conditions during vinification [15]. Hence, important quantities of phenolic compounds still remain in the wine by-products and there is great interest to obtain potentially bioactive phenolic compounds from this type of grape by-product [16,17,18]. Grape seeds are an abundant source of proanthocyanidins with varying degrees of polymerization, and these can become nutraceuticals in numerous products.

Grape seed proanthocyanidin extract can neutralize free radicals, protect the over-oxidative damage caused by free radicals [19,20], and reduce the incidence of a range of diseases caused by free radicals, such as myocardial infarction, atherosclerosis, and drug-induced liver and kidney injury. Moreover, they have antithrombotic, antitumor, anti-mutagenic, anti-radiation-damage, and antifatigue effects [21,22].

Experimental studies [23] indicate that grape polyphenols could reduce atherosclerosis by a number of mechanisms such as inhibition of oxidation of low density lipoprotein (LDL) affecting cellular redox state, improvement of the endothelial function, lowering of blood pressure, inhibition of platelet aggregation, reduction of inflammation, and activation of novel proteins preventing cell senescence in that way.

A wide variety of flavonoids are distributed in vegetables. Onion bulbs (*Allium cepa* L.) are among the richest sources of dietary flavonoids and contribute, to a large extent, to the overall intake of flavonoids. Flavonoids could continue to exert significant effects on inflammation, cardiovascular diseases, and cancer [24]. Slimestad et al. [25] reported on more than 50 flavonoids identified in pigmented scales of onions. Flavonols are the main flavonoids of these pigmented scales of onions and the main flavonols are based on quercetin (3,5,7,3′,4′-pentahydroxyflavone).

Anthocyanins, carotenoids, betalains, lycopenes, and leucoanthocyanidin represent the major groups of colored phenolic compounds in fruit and vegetable residues. They are powerful antioxidants and may possess pharmacological properties, hence making them attractive as “functional foods” for health.

Most of the 200,000 tons of red beet produced in Western Europe annually are consumed as vegetables [1]. The remainder is processed into juice, coloring foodstuff, and food colorant. The latter is commonly known as beetroot red. The pomace from the juice industry accounts for 15%–30% of the raw material and although it is still rich in betalains, it is disposed of as feed or manure [1]. The colored fraction consists of betacyanins and betaxanthins. Moreover, the phenolic portion of the peel shows l-tryptophane, *p*-coumaric, and ferulic acids, as well as cyclodopaglucoside derivatives. Beets are ranked among the 10 most potent vegetables with respect to antioxidant capacity, with a total phenolic content of 50–60 mmol/g dry weight [1].

Lycopene is the principal carotenoid, causing the characteristic red hue of tomatoes. Skin being rich in lycopene, is an important component of waste originating from tomato paste manufacturing plants. Several epidemiological studies have reported that lycopene-rich diets have beneficial effects on human health. Hence, tomatoes and tomato products could lead to the prevention of cardiovascular disease and protection against certain types of cancer [13].

## 3. Examples of Fruit Processing for Recovery of High-Value Products

The comparative presentation of the various fruit waste treatment methodologies showed that although anaerobic digestion stands for the most environmentally friendly technique, it required longer treatment time in conjunction with its weakness to deal with elemental contaminants, making the employment of a second alternative technique which could be a membrane process imperative (low energy cost, reliability, reduced capital cost). Biogas production appears to be another promising and energy effective waste treatment method [26].

Typically, different types of flavonoids, phenolic acids, and gallo- and ellagitannins form the bioactive (antimicrobial and/or antioxidant) phenols in fruit-based extracts are mostly nonvolatile and concentrated in the skin and seeds.

The shelf life of fresh fish and meat, transported over long distances, could be extended by using plant-based extracts to control spoilage bacteria. Widsten et al. [27] identified plant-based extracts that effectively suppress the main spoilage bacteria of chilled fish and lamb and assessed their antioxidant capacity. The phenolic compounds in woodbased tannins and extracts isolated from by-products of the fruit processing industry were identified and/or quantified. The total phenol content was strongly associated with higher antibacterial activity against several fish and lamb spoilage bacteria as shown by zone of inhibition and minimum inhibitory concentration assays. It was also associated with greater antioxidant capacity in the DPPH (2,2-diphenyl-1-picrylhydrazyl) radical assay. Mango seed extract and tannic acid containing mostly polygalloyl glucose type phenols were the most promising candidates for antibacterial packaging or antioxidant dietary supplements.

### 3.1. Grapes and Grape Skin Pulp

Thousands of tons of grape skin pulp (GSP) are produced annually as a by-product of the wine industry. A small proportion of this material is used as animal feed, but most of it is disposed of as waste. Hot water extracts of grape skin pulp (GSPE) can serve as a good substrate for fermentation with *Aureobasidium pullulans* (a yeast like fungus) for the production of pullulan (α-glucan). Pullulan is a commercially important polysaccharide with many industrial applications. This paper reports on the fermentation parameters for pullulan production from GSPE [28].

Several agro-industrial wastes and by-products have been reported as potentially suitable substrates for pullulan synthesis. These include peat hydrolyzate [29], spent sulfite liquor [30], olive oil wastes, molasses [31,32], fuel ethanol by-products [33], inulin hydrolyzate [34], jiggery [35], hydrolyzed potato starch waste [36] and carob pod extract [37].

Grape skins, grape seeds and the stems, which are by-products of the wine production, are separated and sold to distilleries. These grape by-products are then processed into an array of products, including wine alcohol, grape skin extracts, colors, and tannins.

Grape seeds are derivatives of these by-products with a high content of polyphenols and oils, which make them a valuable source for the production of antioxidants and cooking oil (grape seed oil). The grape by-products, the ‘‘marc’’ i.e., the seeds, stems, and skins (the solids that remain after pressing the grapes during wine production) need to be washed for use in distilleries and distillation. Two methods of extraction of crude oil exist. One is mechanical extraction, where the oil is extracted from the seed, bean or fruit, and then removed through a process known as solvent extraction.

The marc can be separated from the must through mechanical methods such as cold pressing. The marc recovered from the pressing process can be recovered as secondary raw material for the production of grappa, which is the grape marc. Otherwise, marc can be disposed of in a landfill as a nonhazardous waste.

From grape seeds, it is possible to obtain a valuable oil with the greatest amount of linoleic acid (70%), which exerts an anticholesterolic action and contains antioxidants and vitamins [38].

Grape pomace is a by-product rich in phenolic compounds playing a key role as an antioxidant and due to its high fiber concentration, it could be used as an alternative ingredient to partially replace forage in the diet of small ruminants. Manso et al. [39] evaluated the effect of dietary supplementation of vitamin E or different doses of grape pomace associated with linseed oil on milk fatty acid profile, composition, and yield. Forty-eight Churra ewes were fed with experimental diets consisting of a total mixed ration (TMR) containing 2.7% [on a dry matter (DM) basis] of linseed oil, forage, and concentrate at a 40:60 ratio. Ewes were assigned to one of four treatments: control (without grape pomace), vitamin E (with 500 mg/kg of TMR of vitamin E), grape pomace-5 (5 g/100 g of TMR of DM of grape pomace), and grape pomace-10 (10 g/100 g of TMR of DM of grape pomace). Experimental diets did not affect DM intake and milk yield and composition. The vitamin E supplementation had only a moderate effect on milk concentration of fatty acids (increase in α-linolenic acid and 16:0 and decrease in *cis-*9 18:1). Percentages of total saturated, monounsaturated, and polyunsaturated fatty acids were not affected by grape pomace supplementation. Levels of α-linolenic acid reached about 1% of total fatty acids due to the presence of linseed oil in the diets, were not modified with vitamin E, and remained unaltered following treatment with grape pomace-5 and -10. Linoleic acid was increased by the highest dose of grape pomace.

Another reason why grape pomaces (GPs) are characterized by high contents of phenolics is due to an incomplete extraction during the winemaking process. As already reported, these phenolics are quite beneficial to human health due to their antioxidant activity and antimicrobial, antiviral, and anti-inflammatory properties. Therefore, GPs constitute an inexpensive source for the extraction of phytochemicals that can be used in the pharmaceutical, cosmetic, and food industries. Hence, efforts have been made to use GP in different fields of industry. Efficient extraction techniques need to be employed and sensitive and selective analytical methods need to be tried in order to achieve good recoveries of compounds and characterization of phenolic extracts. Fontana et al. [40] summarized the most recent developments in the extraction of polyphenols from GPs. Furthermore, the techniques used for characterization of extracts were explained, with emphasis on sample preparation, separation, and analysis of phenolics and applications of GP extracts in diverse biotechnological fields were also discussed.

### 3.2. Cherry Seeds

About 25% of the total product is intended for syrup, alcohol cherries, candied fruit, juices, jams and liqueurs manufacturing.

Cherry seeds’ waste is primarily destined to landfill (European waste code 020304), and corresponds to residues from the production of canned food unsuitable for consumption or further processing. Cherry seeds along with other types of waste with low moisture content, represent the most suitable biomass for the thermo-energetic application.

Seeds could be transformed into briquettes, which can be easy to store and transport, and may feed stoves and domestic boilers. Simultaneously, they can ensure the maintenance of the heat in the firebox and a low production of combustion ashes. These residues can also be used in anaerobic digestion [38].

### 3.3. Banana

Banana plant biomass waste, viz. pseudostem (BPS) and rhizome (BR), contribute 30.81 and 12.67 per cent, respectively. A negligible percentage of these is used for fresh consumption, otherwise they are wasted and incinerated. In order to utilize these bio-wastes in a bioactive perspective, nutritional and nutraceutical components were studied from the juices and its Ready-To-Serve (RTS) beverage. BR juice showed higher total phenolic content (TPC) and total flavonoid content (TFC) of 341.44 mg GAE and 87.60 mg CE/200 mL, respectively when compared to BPS juice. Moreover, it exhibited high antioxidant activity (AOA) in all the assays tested, viz. DPPH radical scavenging activity (82.93%), superoxide radical scavenging activity (49.45%), metal chelation activity (48.32%) total reducing power (0.846 OD at 700 nm) and total antioxidant capacity (928 mmol ascorbic acid equivalents). A high quantity of polyphenols present in BR juice resulted in high AOA. Strong positive correlations were observed between the TPC and TFC of BPS and BR juice with AOA assays. Among the different concentrations of RTS beverages, 25% BPS juice and 20% BR juice with 15 °brix Total soluble solids (TSS) and 0.3% acidity were judged as the best ones by sensory panelists. Thus, BPS and BR juice can be effectively used to produce new generation functional beverages [41].

BPS appears to be a rich source of fiber, total carbohydrate and cellulose. Manimehalai [42] observed moisture, protein, fat, minerals, fiber and carbohydrates content of BPS of 93.1, 0.3, 0.03, 1.04, 0.68 and 1.20 g/100 g, respectively.

The nutritive value of BR includes moisture, protein, fat, minerals, crude fiber, carbohydrates, calcium, phosphorus, iron, and energy of 85.1, 0.4, 0.2, 1.4, 1.1, 11.8, 0.025, 0.010, 0.0011 g and 51 kcal/100 g of edible portion, respectively [43].

### 3.4. Pineapple

Pineapple (*Ananas comosus* (L.) Merr. var. *Mauritius*), an edible fruit native to Central and South America, is grown in several tropical and subtropical countries, including India. The total pineapple production worldwide is 16 to 18 million tons [44]. The ethanolic extract of pineapple leaves has been explored for anti-diabetic and dyslipidaemia properties [45,46]. Residual fiber and skin, constituting approximately 30%–35% of pineapple fruit, are usually discarded as low-value by-products. The peel and residual pulp of pineapple fruit are collectively referred to as pineapple fruit residue. Different phytochemicals have been previously extracted from pineapple fruit residue [47]. However, there has been no systematic evaluation of its use as a nutraceutical property against diabetes. Riya et al. [48] reported the effects of pineapple ethyl acetate extract (PAE) and pineapple methanol extract (PAM) and studied them for their anti-diabetic potential against key targets such as carbohydrate digestive enzyme inhibition, DNA damage protection, anti-glycation activity and glitazone-like property.

### 3.5. Apple Pomace

Apple pomace, a left-over biomass generated in huge quantities in fruit-processing industries, contains peel, seeds, core, calyx and stem tissues [49]. Due to its significantly high moisture content and the presence of fermentable carbohydrates, it is sensitive to microbial decomposition [50]. Moreover, its disposal in open areas leads to serious environmental problems. It is mostly used for extraction of pectin and animal feed production. Pomace can be utilized either in a waste reduction strategy or for the development of a high-value-added product, or for both. [51]. Apple pomace has been considered a valuable biomass for the extraction/production of products such as pectin [52], organic acids [53], protein-enriched feeds [54], aroma compounds [55], enzymes [56], natural antioxidants [57], and edible fibers. [58]. It contains 4%–5% seeds, a significant part after the peel, and can be used for oil extraction [59]. Apple pomace, including seeds, is rich in polyphenols. Apple seeds contain phloridzin, a major phenolic compound. Phloretin-2′-xyloglucoside, 5-caffeoylquinic acid (chlorogenic acid), *p-*coumaroylquinic acid, (−)epicatechin, are compounds, which have been found in apple seed extracts [60].

Walia et al. [61] used apple seeds separated from industrial pomace for the extraction of oil. The fatty acid composition, physicochemical and antioxidant as well as in vitro anticancer properties of extracted oil were studied to assess its suitability in food and therapeutic applications. The fatty acid composition of seed oil revealed high concentrations of oleic (46.50%) and linoleic acid (43.81%). It had a high iodine (121.8 g·iodine 100 g^−1^) and saponification value (184.91 mg·KOH·g^−1^ oil). The acid value, refractive index and relative density were 4.28 mg·KOH·g^−1^, 1.47 and 0.97 mg·mL^−1^, respectively. The antioxidant potential (IC50) of apple seed oil was 40.06 μg·mL^−1^. Cytotoxicity of apple seed oil against CHOK1, SiHa and A549 cancer cell lines ranged between 0.5% ± 0.06% and 88.6% ± 0.3%.

### 3.6. Coffee Fruit

Chlorogenic acids belong to phytochemicals, the most common being caffeic acid, ferulic acid, and *p*-coumaric acid which form esters with quinic acid. [62,63,64]. They belong to hydroxycinnamic acids, a type of phenolic compound with a C6–C3 skeleton. The chlorogenic acid content of the coffee beverage has been extensively studied [65], and the transformation of hydroxycinnamates during coffee roasting has been the subject of a number of research papers [66].

In contrast to green and roasted coffee beans and instant coffees, there is not much work being carried out on the chlorogenic acid contents of soluble green coffee extracts [67].

The whole coffee fruits, or cherries (with seed intact), may provide a broad range of phenolic and polyphenolic compounds not found in the beans. In addition, the husks of the coffee fruits could be a valuable source of polyphenolic compounds and these are normally discarded as waste.

Air-dried whole coffee fruits, beans, and husks from China, India, and Mexico were analyzed for their chlorogenic acids (CGA), caffeine, and polyphenolic content. Analysis was carried out by HPLC and Orbitrap exact mass spectrometry and the total phenol, total flavonol, and antioxidant capacity were measured. The hydroxycinnamate profile consisted of caffeoylquinic acids, feruloyquinic acids, dicaffeoylquinic acids, and caffeoyl-feruloylquinic acids. A range of flavan-3-ols as well as flavonol conjugates were detected. The CGA content was similar for both Mexican and Indian coffee fruits but was much lower in the samples from China. The highest levels of flavan-3-ols were found in the Indian samples, whereas the Mexican samples contained the highest flavonols. Amounts of CGAs in the beans were similar to those in the whole fruits, but flavan-3-ols and flavonols were not detected. The husks contained the same range of polyphenols as those in the whole fruits. The highest levels of caffeine were found in the Robusta samples [68].

### 3.7. Litchi

Litchi (*Litchi chinensis* Sonn.) is a subtropical fruit with high commercial value, widespread in the south of China. Since 2005, its cultivated area was more than 6 × 10^5^ ha, and its yield was more than 1.3 × 10^6^ tons per year. It has been shown that the processing by-products of litchi fruit, such as flowers, pericarp, and seeds, all have antioxidant properties [69,70,71]. It was also reported that the pericarp of litchi had been used as a traditional medicine with hemostatic and acesodyne functions in ancient times [72]. Litchi pericarp procyanidin extract was effective for the prevention and treatment of hyperuricemia and/or gout [73] and showed a protective effect against cardiovascular diseases. However, the pericarp of litchi, which accounts for 15% of the fresh weight of the fruit, becomes desiccated and turns brown at ambient temperature within 2 or 3 days post-harvest and is often thrown away as waste [74].

A-type procyanidins, being considered a natural dietary supplement due to their high biological activity in vivo, are derived from litchi processing waste from *Litchi chinensis* pericarp. Litchi pericarp oligomeric procyanidins (LPOPCs) did not selectively modify the growth of *Streptococcus thermophilus* and *Lactobacillus casei*-01 at concentrations of 0.25 and 0.5 mg/mL, and it was demonstrated that the two strains could transform procyanidins during their log period of growth by two different pathways. *S. thermophilus* was able to metabolize procyanidin A2 to its isomer, and *L. casei* could decompose flavan-3-ols into 3,4-hydroxyphenylacetic acid, 4-hydroxyphenylpropionic acid, m-coumaric acid, and pcoumaric acid. The total antioxidant capability (T-AOC) of LPOPCs before and after microbial incubation was estimated. Results suggested that probiotic bacteria bioconversion is a feasible and efficient method to convert litchi pericarp procyanidins to a more effective antioxidant agent [75].

### 3.8. Olive Fruit

The most abundant polyphenols identified in olive leaf extracts have been oleuropein, hydroxytyrosol, verbascoside, apigenin-7-glucoside, and luteolin-7-glucoside [76]. These compounds confer bioactive properties on the olive leaf extracts, such as antioxidant [76], antimicrobial [77], and antitumor capacities [78]. Moreover, they are able to reduce the risk of coronary heart disease [79].

Not only do olive leaves appear as a by-product during oil processing in the olive oil industries (10% of the total weight of the olives), but they are also a residue of olive tree pruning [80].

Ahmad-Qasem et al. [81] assessed the effect of processing conditions (drying and extraction) of olive leaves on the extract’s bioaccessibility. Thus, extracts obtained from dried olive leaves (hot air drying at 70 and 120 °C or freeze-drying) by means of conventional or ultrasound-assisted extraction were subjected to in vitro digestion. Antioxidant capacity, total phenolic content, and HPLC-DAD/MS/MS analyses were carried out during digestion. Dehydration used for the olive leaves did not have a meaningful effect on bioaccessibility. The digestion process significantly (*p* < 0.05) affected the composition of the extracts. Oleuropein and verbascoside were quite resistant to gastric digestion but were largely degraded in the intestinal phase. Nevertheless, luteolin-7-*O*-glucoside was the most stable polyphenol during the in vitro simulation (43% bioaccessibility). Therefore, this compound might be used in studies of the bioactivity of olive leaf extracts.

Production of Extra Virgin Olive Oil (EVOO) is also associated with the generation of large quantities of wastes [82,83], and it is associated with the loss of olive polyphenols in olive oil by-products. Qualitative and quantitative characterization of the phenolic profile in these wastes has been determined extensively [84].

Frankel et al. [85], in their review, described the olive oil production process to obtain extra virgin olive oil (EVOO) enriched in polyphenol and by-products generated as sources of antioxidants. EVOO is obtained exclusively by mechanical and physical processes including collecting, washing, and crushing of olives, malaxation of olive paste, centrifugation, storage, and filtration.

The effect of each step is discussed to minimize losses of polyphenols from large quantities of wastes. Phenolic compounds including phenolic acids, alcohols, secoiridoids, lignans, and flavonoids are characterized in olive oil mill wastewater, olive pomace, storage by-products, and filter cake. Different industrial pilot plant processes are developed to recover phenolic compounds from olive oil by-products with antioxidant and bioactive properties. The technological information added in this review will help olive oil producers to improve EVOO quality and establish new processes to obtain valuable extracts enriched in polyphenols from by-products with food ingredient applications.

Olive mill wastewater (OMWW), the main waste product of olive oil extraction process, was investigated as a source of polysaccharides. The yield of alcohol insoluble residue (AIR) was 20.5% based on the dry matter of OMWW. Extraction with water gave water soluble (WSF) and insoluble (WIF) fractions from AIR with yields of 13.3% (*w*/*w*) and 3.7% (*w*/*w*) based on the dry matter, respectively. Chemical composition and monosaccharide analysis showed that glucose was the main monosaccharide of these extracts in addition to galactose, arabinose, rhamnose, and galacturonic acid. Prebiotic and antioxidant activities of polysaccharidic fractions from OMWW were evaluated by Nadour et al. [86]. Results showed their scavenging capacity toward the 2,2′-diphenyl-1-picrylhydrazyle (DPPH) (IC50 value of 89.43 μg/mL) and hydroxyl radicals (IC50 value of 158.70 μg/mL), resistance toward artificial human gastric juice, and ability to be fermented by Lactobacilli strains.

### 3.9. Winery and Olive Mill Waste

Winery and olive mill wastes might be regarded as potential resources for Solid substrate fermented (SSF) substrates, due to their simple and complex sugars and other nutrients [87]. Note, vine shoot trimmings, and olive pomace are lignocellulosic wastes, which were not exploited in the past. Lignocellulosic wastes are attractive feedstocks for microbial enzyme production [88]. Valuable products such as biofuels, chemicals, and animal feed lately have been made by the conversion of lignocellulosic wastes [89].

They may also be a suitable substrate to induce fungal cellulase production in SSF because their major component is cellulose.

Wineries and olive oil industries are dominant agro-industrial activities in southern European regions. Olive pomace, exhausted grape marc, and vine shoot trimmings are lignocellulosic residues generated by these industries, which could be valued biotechnologically. Salgado et al. [90] used these residues as substrates to produce cellulases and xylanases through solid-state fermentation using *Aspergillus uvarum* MUM 08.01. For that, two factorial designs (3^2^) were first planned to optimize substrate composition, temperature, and initial moisture level. Subsequently, the kinetics of cellulolytic enzyme production, fungal growth, and fermented solid were characterized. Finally, the process was performed in a packed-bed bioreactor. The results showed that cellulase activity improved with the optimization processes, reaching 33.56 U/g, and with the packed-bed bioreactor aeration of 0.2 L/min, reaching 38.51 U/g. The composition of fermented solids indicated their potential use for animal feed because cellulose, hemicellulose, lignin, and phenolic compounds were partially degraded 28.08%, 10.78%, 13.3%, and 28.32%, respectively; crude protein was increased from 8.47% to 17.08%, and the mineral contents met the requirements of main livestock [90].

### 3.10. Pomegranate

The non-edible fractions of pomegranate fruit and tree, which are waste materials (i.e., peel, seeds, flowers, bark, buds and leaves), contain even higher amounts of specific nutritionally valuable and biologically active components compared to the edible fruit [91,92,93].

Hydrolysable polyphenols in peel (PoP), specifically ellagitannins, are the most active antioxidants amongst the tannins contained. These compounds (ellagic acid, punicalagin, punicalin and gallagic acid) have been shown to hold raised antioxidant and pleiotropic biological activities and notably, to act synergistically together [94].

Akhtar et al. [95] focused on the nutritional, functional and anti-infective properties of pomegranate (*Punica granatum* L.) peel (PoP) and peel extract (PoPx) and on their applications as food additives, functional food ingredients or biologically active components in nutraceutical preparations. The biomolecules available in PoP and PoPx due to their well-known ethnomedical relevance and chemical features have been proposed, as substitutes of synthetic food additives, as nutraceuticals and chemopreventive agents. However, PoP and PoPx are not yet considered as ingredients of choice in food systems due to their astringency and anti-nutritional properties. Indeed, the nutritional and nutraceutical potential of PoP and PoPx seems to be still underestimated and not investigated thoroughly. Their review meticulously covers the applications of PoP and PoPx components in various food products as food preservatives, stabilizers, supplements, prebiotics and quality enhancers. Further investigations in toxicological and sensory aspects of PoP and PoPx should be encouraged to fully exploit the health promoting and technical/economic potential of these waste materials as food supplements.

Pomegranate is increasingly consumed as various processed products, such as juices, wines, jams, jellies and extracts. In pomegranate juice processing, 1 ton of fresh fruit generates 669 kg by-product—pomegranate marc—containing 78% peel and 22% seeds, based on a previous study [96]. They also found that the peel of pomegranate marc had a high level of phenolics (content of 201 g·kg^−1^ based on dry weight).

Liquid extracts from pomegranate peel have the potential for use as natural antioxidant products. Qu et al. [97] investigated the quality changes of liquid extracts before and after thermal treatment during sterilization and storage. Liquid pomegranate peel extracts were prepared, sterilized under ultra-high temperature (UHT) at 121 °C for 10 s and then stored at three temperatures (4, 25 and 37 °C) for up to 180 days. The industrial, color, UV-visible spectrum profile and antioxidant (phenolics) characteristics were measured.

Thermal sterilization treatment showed no negative effects on the industrial, color, spectral and antioxidant characteristics of the extracts. After 180 days, the extracts stored at 4 °C retained 67% of the initial total soluble phenolic content and 58% of the original scavenging activity. The major antioxidant components in the extracts (stored at 4 °C for 180 days) were gallic acid, punicalagin A, punicalagin B and ellagic acid wirh concentrations of 19.3, 197.2, 221.1 and 92.4 mg·L^−1^, respectively.

### 3.11. Jabuticaba

There are only a few studies concerning the peel fraction of jabuticaba [98]. Jabuticaba (*Myrciaria cauliflora*. Mart) is a highly perishable fruit native to Brazil, which is consumed both fresh and industrially processed in the form of juices, jams, wines and distilled liqueurs. This processing generates a large amount of waste by-products, which represent approximately 50% of the fruit weight.

Valuable bioactive compounds, that could be used as nutraceuticals or functional ingredients, could be obtained by these by-products. In a study [99], fermented and non-fermented jabuticaba pomaces were studied regarding their hydrophilic and lipophilic compounds, as well as their antioxidant properties, including soluble sugars, organic acids and tocopherols (using techniques such as high performance liquid chromatography coupled to a refraction index, diode array and fluorescence detector, respectively); phenolics and anthocyanins (using liquid chromatography coupled to diode array detection, and mass spectrometry with electrospray ionization); and fatty acids (using gas–liquid chromatography with flame ionization detection). The analytical data demonstrated that jabuticaba pomaces are a rich source of bioactive compounds such as tocopherols, polyunsaturated fatty acids and phenolic compounds (namely hydrolysable tannins and anthocyanins) with antioxidant potential. Therefore, jabuticaba pomace may have good potential as a functional ingredient in the fabrication of human foods and animal feed.

In Table 1 phenolic constituents as nutraceuticals from different fruit residues are well described.

### 3.12. Citrus Pomace

A large amount of waste is generated by the *Citrus* juice industry and this waste is a good source of pectins, essential oils, polyphenols, flavonoids. Moreover, the citrus flavonoids—naringin, and naringenin—were found to have potential applications in the prevention of atherosclerosis [12]. Bioflavonoids such as hesperidin (from orange peel), naringin, eriocitrin, and narirutin can be found in citrus peel residues [13,105].

Residues of citrus juice production are a source of dried pulp and molasses, fiber-pectin, cold-pressed oils, essences, d-limonene, juice pulps and pulp wash, ethanol, seed oil, pectin, limonoids [1].

## 4. Examples of Vegetable Processing/Potato Processing and Others (Grains, Cereals, Nuts) for Recovery of High Value Products

Vegetable industries have been considered responsible for a great amount of pollution, hence, there has been a strong need for the optimization of vegetable waste treatment systems. The comparative presentation of the various vegetable waste treatment methodologies showed that although anaerobic digestion stands for the most environmentally friendly technique, it required longer treatment time in conjunction with its weakness to deal with elemental contaminants, making imperative the employment of a second alternative technique which could either be a membrane process (low energy cost, reliability, reduced capital cost) or a coagulation/flocculation method because of its low cost and high effectiveness. Biogas production appears to be another promising and energy effective waste treatment method [113].

Vegetable residues can be used as a source of potential phytochemicals. Olive pomace is used as a nematode controlling agent for tomatoes [114]; citrus waste streams are used in horticulture [115] and mandarin peel flavonoids are interesting due to their fungistatic activity [116] which may be applied to naturally protect vegetables and fruits from molding.

The raw material mostly used is carrot pomace [117,118,119,120,121], followed by citrus waste [115,122], grape or apple pomace [119,123,124,125,126], sugar beet pomace [127,128,129], orange, mango and apple peel [130], mango kernel flour [131] (as a wheat flour substitute), potato peel [132], sugarcane bagasse [133] or mixtures of oat, rice, corn hulls and pea pods [134]. They are applied in pie fillings [124], crackers [124,135], bread [117,133], cookies [129,133], beverages [118,121,122,136], jam [137], and cakes, dressings and pickles [138].

Carrot pomace works as a stabilizer in bread and bakery goods, as well as in pastry, cereals and dairy products. Some of its functional properties include crude fiber, richness in provitamins, color and natural acids, sourdough substitution in bread, acidifying agent, preservative or antioxidant in several food products [117,119,126,138,139].

In beverages, carrot pomace or citrus waste will stabilize the natural color, improve the vitamin and fiber content, enhance the viscosity (mouthfeel) [118,121,136], and enrich or adjust the cloudy appearance [122]. The organoleptic and chemical properties offer a widespread use in healthy and functional drinks and selected fruit juices.

### 4.1. Potato Peel Waste

The problem of potato peel waste (PPW) management causes great concern to the potato industries in Europe and an integrated and environmental friendly solution is yet to be found and is under investigation. The potato peel is a zero-value waste of the potato processing plants.

While consumption of potatoes has decreased, processed products such as French fries, chips, and puree have experienced growth [140]. Losses caused by potato peeling range from 15% to 40%, their amount depending on the process applied, i.e., steam, abrasion or lye peeling [1].

PPW contains sufficient quantities of starch, cellulose, hemicellulose and fermentable sugars and can serve as an ethanol feedstock. A number of batches of PPW were hydrolyzed with various enzymes or acid, and then fermented by *Saccharomyces cerevisae* var. *bayanus* to determine fermentability and ethanol production.

Enzymatic hydrolysis with a combination of three enzymes, released 18.5 g·L^−1^ reducing sugar and produced 7.6 g·L^−1^ of ethanol after fermentation. The results demonstrate that PPW, a by-product of the potato industry features a high potential for ethanol production [141].

The potato processing industries producing chips, peeled potatoes and wedges, etc., generate a large amount of starchy wastewater which contains high concentrations of chemical oxygen demand (COD), 5-day biochemical oxygen demand (BOD5) and suspended solids (SS). In most cases, the potato processing wastewater without treatment is directed to the landfill which causes serious environmental threats. The European Union (EU) Landfill Directive (1999/31/EC) has stipulated that Member States should divert 65% of 1995 biowaste levels from landfill by 2020. Hence, different methods, such as aerobic biotechnologies, lagoons, land applications and electro-coagulation, have been used or developed to treat the potato processing wastewater. Nevertheless, those technologies do not recover any high value products [142,143]. Few studies focused on the production of value-added products such as biogas, lactic acid, and microbial proteins, but did not study in detail the simultaneous removal of potential nutrients such as total nitrogen (TN) and total phosphorus (TP) from wastewater [144]. To date, literature on uses of potato processing wastewater for microbial lipid production is very sparse. Therefore, this study by Muniraj et al. [145] investigated the bioconversion of potato processing wastewater into lipid-rich biomass using two oleaginous fungi—*Aspergillus flavus* I16-3 and *Mucor rouxii—*with simultaneous removal of nitrogen and phosphorus from wastewater.

Biochemistry and physiology of two oleaginous fungi, *A. flavus* I16-3 and *M. rouxii*, on lipid accumulation showed the two fungi grew well and efficiently utilized the starch in wastewater. On average (*p* < 0.05), 2.8 and 3.6 g·L^−1^ of lipids were produced by *A. flavus* I16-3 and *M. rouxii*, respectively, with maximum g-linolenic acid (GLA) yields of 60 and 100 mg·L^−1^. The addition of nutrients to raw wastewater significantly improved (*p* < 0.05) the lipid and GLA yields. *A. flavus* I16-3 and *M. rouxii*, produced 3.5 and 4.2 g·L^−1^ of lipids, and 100 and 140 mg·L^−1^ of GLA, respectively. In addition, the wastewater was efficiently treated, with soluble chemical oxygen demand, total soluble nitrogen and total soluble phosphorus removals up to 60% and 90%, 100% and 98%, and 92% and 81% by *A. flavus* I16-3 and *M. rouxii*, respectively. This study demonstrated an alternative approach to valorize potato-processing wastewater to produce microbial lipids and GLA (nutraceuticals).

### 4.2. Sweet Sorghum

The sweet sorghum contains sufficient quantities of sucrose, glucose, hemicellulose and fermentable sugars and can serve as an ethanol feedstock. Fresh juice extracted by pressure from nine sweet sorghum varieties as well as whole stalks was fermented by *Saccharomyces cerevisiae* var. *Bayanous* for bioethanol production. The juice was used directly, as were whole stalks after previous acidic hydrolysis. The extracted juice varied from 33% to 54% fresh stalks of sweet sorghum and contained 8.9% to 16.4% fermentable sugars. The results showed that for most sweet sorghum varieties, ethanol production was 7 g·L^−1^ and 10 g·L^−1^ ethanol from the fermentation of juice and whole stalks, respectively. The ethanol yield was high and corresponded to up to 70% of the max theoretical yield for the fermentation of juice and 80% for the whole stalks [146,147,148].

### 4.3. Maize Cobs

Maize is a high represented crop in many parts of the World. Serbia belongs to the countries where maize growing is highly represented. It covers about 38% of arable land. More important is that about 90% of maize is on small and medium farms, less than 200 ha [149].

The objective of this work is to test the possibility of the production of bioethanol from maize cobs and estimate the potential yield. This should be the background for the evaluation of applicability of this biomass for bioethanol production.

The released sugars from maize during acidic hydrolysis were 13.35, 22.99 and 23.44 g·L^−1^ for samples 1, 2 and 3, respectively. These sugars were fermented by *S. Cerevisiae var. Bayanus.* At the end of fermentation, the concentration of sugars was very low (approx. 1.5 g·L^−1^ for all samples) whereas the sugars’ consumption was too high.

Bioethanol production was 4.8 g·L^−1^ for variety no. 1, whereas for the other two it was much higher, varying between 8.78 and 7.90 g·L^−1^ (nο. 2 and nο. 3, respectively). The corresponding yield Y p/_S_ (g ethanol/g consumed sugars) varied between 0.360 (or 36.0%) for sample nο. 3, and 0.412 (or 41.2%) for sample nο. 2. This yield corresponds to 72.04% of the max. theoretical yield for sample nο. 3 and 82.44% for sample nο. 2 [150].

### 4.4. Xylooligosaccharides and Brewer’s Spent Grain (BSG)

Inulin, lactulose, galactooligosaccharides (GalOS), or fructooligosaccharides (FOS) are already considered prebiotics and they are commercially available. However, others such as xylooligosaccharides (XOS), considered as being nondigestible oligosaccharides by gastric or pancreatic enzymes, are gaining importance in this field. XOS are reaching the colon intact after oral intake but can be used by a selected group of beneficial gut microflora for exhibiting several physiological changes [151,152,153].

XOS can be obtained from plant biomass by chemical treatments, autohydrolysis, enzymatic hydrolysis, or a combination of these processes. In this context, Brewer’s spent grain (BSG) is a fiber-rich by-product generated in beer factories at high volume and low cost [154,155,156], and could be used as raw material for AXOS production due to the presence of approx. 42% *w/w* of polymeric/oligomeric material, mainly composed of xylose [157].

The potential of Brewer’s spent grain (BSG) samples as a raw material in order to obtain a mixture of arabinoxylooligosaccharides (AXOS) suitable to be used as prebiotics for elderly [158] was assessed by means of two-step aqueous processing (starch extraction and autohydrolysis). Following hydrothermal treatment, the liquors were refined by a sequence of purification and conditioning steps including membrane filtration, enzymatic hydrolysis, and ion exchange. The presence of both substituted (degree of polimerization (DP) = 2–10) and unsubstituted (DP = 2–16) oligosaccharides made up of xylose and arabinose (AXOS) were confirmed in purified mixtures (in which total Solids content (OS) content = 84% *w/w*) by using chromatographic techniques and matrix-assisted laser desorption/ionization-time-of-flight mass spectrometry (MALDI-TOF MS). Finally, AXOS were evaluated for their prebiotic activity by in vitro fermentation assays using fecal inocula from elderly people, demonstrating that AXOS were slightly better substrates than FOS, in terms of bacterial population shifts as in the production of short chain fatty acids (SCFA).

### 4.5. Pistacchio Hulls

The hull (soft epicarp of pistachio nuts which clings tightly to the hard inner shell) is the largest waste product (accounting for on average between 35% and 45%) of the pistachio industry. It has a reddish/yellow color during maturation, becoming rosy-light yellow when ripe. In ripe nuts, it can be peeled off easily. There is very little information regarding its use, biological properties and health promoting compounds, although it is considered a promising source of primary (mainly protein and fat) and secondary (mainly phenol derivatives) metabolites, as well as minerals, vitamins and essential oil [159,160].

Rajaei et al. [161] recently analyzed its anti-microbial and antimutagenicity activities and Goli et al. [160] investigated its total phenol amount and effectiveness at retarding oil deterioration at 60 °C.

Every year, tons of pistachio hulls are separated and eliminated, as waste products, from pistachio seeds. Barreca et al. [162] extracted the hulls of ripe pistachios with two organic solvents (ethanol and methanol) and characterized them for phenolic composition, antioxidant power and cytoprotective activity. RP-HPLC-DAD-FLU separation enabled them to identify 20 derivatives, including by far the most abundant gallic acid, 4-hydroxybenzoic acid, protocatechuic acid, naringin, eriodictyol-7-*O*-glucoside, isorhamnetin-7-*O*-glucoside, quercetin-3-*O*-rutinoside, isorhamnetin-3-*O*-glucoside and catechin.

Methanol extraction gave the highest yields for all classes of compounds and presented a higher scavenging activity in all the antioxidant assays performed. The same was found for cytoprotective activity on lymphocytes, lipid peroxidation and protein degradation. These findings highlight the strong antioxidant and cytoprotective activity of the extract components, and illustrate how a waste product can be used as a source of nutraceuticals to employ in the manufacturing industry [162].

### 4.6. Pumpkin

The pumpkins (*Cucurbita* sp.) were domesticated in the New World and planted long ago by the Amerindians. *Cucurbita moschata* is the most important species in Tropical America, due to the area where it has expanded and its variability, and has Central America and Mexico as its center of diversity. *C. maxima* comes from South America, where it was cultivated in Pre-Columbian times, and is considered one of the oldest species.

The seeds of pumpkins are promising sources of lipid, protein, and ash. Studies have shown that they found 31% to 39% and 24% to 42% of lipids in seeds of *C. moschata* and *maxima* pumpkin species, respectively. They are also rich in mono- and polyunsaturated fatty acids, and have low levels of sugars, starches, and other substances still unknown [163].

The seeds of *Cucurbita* sp. have been used for a long time in Chinese medicine, with reports on combating intestinal parasites and treating biliary vesicle and prostate problems.

In addition, nongerminated seeds have a hypoglycemic effect; they also have antioxidant, anticancer, and anti-inflammatory properties [164].

Seeds, as agro-industrial residues, can be used as a source of macronutrients and/or raw material for extraction of vegetable oils, since they present great quantities of bioactive compounds. This study by [165] aimed to characterize the lipid fractions and the seeds of pumpkin (*Cucurbita* sp.) varieties Nova Caravela, Mini Paulista, Menina Brasileira, and Moranga de Mesa, aiming at using them in food. The chemical composition of the seeds was performed according to the official methods of American Oil Chemists’ Society and Association of Official Analytical Chemists. Total carotenoids and phenolic compounds were determined by spectrophotometry, while the levels of tocopherols were analyzed by high efficiency liquid chromatography. It was noted that the seeds contain high amounts of macronutrients that are essential for the functioning of the human organism. Mini Paulista and Menina Brasileira pumpkin varieties presented significant amounts of total carotenoids, 26.80 and 26.03 μg/g, respectively. Mini Paulista and Nova Caravela pumpkin varieties showed high amounts of total phenolic compounds in the lipid fractions and in the seeds. It was also found that *γ* -tocopherol is the isomer with the highest concentration in the lipid fractions and in the seeds, mainly in Menina Brasileira. Finally, the consumption of these seeds and the use of lipid fractions provide compounds that are beneficial to health and that may be potentially used in food. Moreover, they comprise an alternative, better use of agro-industrial residues [165].

### 4.7. Red Radish

Anthocyanins are water-soluble vacuolar pigments found in most species in plants, responsible for the red, purple, and blue colors of many fruits, vegetables, cereal grains, and flowers [166]. The uses of anthocyanins as colorants have gained much attention from both a legislative point of view action and consumers’ concerns over the use of synthetic additives in foods. Radish anthocyanins have been found to possess high tinctorial power and considerable stability, being a useful alternative to Food and Drug Administration colouring (FD&C) Red No. 40 (allura red) [167], which is one of the most consumed colorants in the world. Radish anthocyanins have been widely applied as natural colorants due to their color characteristics along with health benefits, including antioxidant activities [168,169].

Red radish (*Raphanus* L.) pickles are one type of important fermented vegetable for their colorful and texture properties. Tons of this kind of pickle are annually produced for domestic consumption and for export. Considerable amounts of radish brine are generated as a waste, which represents a challenge for full utilization of radish brines. However, the changes in phytochemical compositions, color properties, and antioxidant activities in the color-rich brine produced during lactic acid fermentation of red radishes are still not clear.

Jing et al. [170] evaluated the dynamic changes in color properties, phenolics, anthocyanin profiles, phenolic acid composition, flavonoids, and antioxidant properties in radish brines during lactic acid fermentation. The results showed that five flavonoids were detected (four anthocyanins and one kaempferol derivative), including pelargonidin-3-digluoside-5-glucoside derivatives acylated with *p*-coumaric acid, ferulic acid, *p*-coumaric and manolic acids, or ferulic and malonic acids. Amounts ranged from 15.5 to 19.3 μg/mL in total monomeric anthocyanins, and kaempferol-3,7-diglycoside (15–30 μg/mL). Gentisic, 4-Hydroxy-benzoic, vanillic, syringic, *p*-coumaric, ferulic, sinapic and salicylic acids were detected in amounts that varied from 70.2 to 92.2 μg/mL, whereas the total phenolic content was 206–220 μg/mL. The change in color of the brine was associated with the accumulation of lactic acid and anthocyanins. The oxygen radical absorbance capacity (ORAC) and Fe^2+^ chelation capacity of radish brines generally decreased, whereas the reducing power measured as ferric reducing antioxidant power (FRAP) values was increased during the fermentation from day 5 to day 14. This study provided information on the phytochemicals and the antioxidative activities of red radish fermentation waste that might lead to further utilization as nutraceuticals or natural colorants.

### 4.8. Rice Bran

Rice bran is a major by-product obtained from the rice polishing process that contains phytosterols such as gamma-oryzanol (1.6%), which is a mixture of ferulic acid (FA) esters of triterpene alcohols such as cycloartenol and 24-methylene cycloartenol [171,172,173]. These triterpene alcohols are now widely used as active ingredients in health foods and as an FA precursor for the production of biovanillin. FA derivatives are active ingredients for the development of therapeutics for the treatment and prevention of cancers and metabolic disorders [174]. FA esters are formed in plant cell walls of monocotyledons such as corn, maize bran, sugar beet, rice endosperm, wheat grain, and barley grain from phenylalanine, tyrosine, and arabinoxylan residues of the cell wall matrix [175,176].

Ferulic acid (FA) is widely used in foods and beverages, as a precursor of vanillin. The growth of lactic acid bacteria (LAB), leads to the biotransformation of FA, which can be converted to other phenolic derivatives, with ferulic acid esterase (FAE) and ferulic acid decarboxylase (FDC) playing significant roles. Kaur et al. [177] aimed at screening a panel of LAB for their ability to release FA from rice bran, an agro waste material. FAE and FDC activities were analyzed for the preliminary screening of various dairy isolates. Two *Pediococcus acidilactici* isolates were selected for studying further the hydrolysis of FA from rice bran and its bioconversion into phenolic derivatives such as 4-ethylphenol, vanillin, vanillic acid, and vanillyl alcohol. *P. acidilactici* M16, a probiotic isolate, has great potential for the production of FA from rice bran and could be considered as a starter culture in the food industry for the production of biovanillin.

### 4.9. Okara

By-products of the soybean food processing represent an important disposal problem for this industry [178]. Okara is an example of a by-product of the soymilk industry. Raw okara is a yellowish-white material consisting of the insoluble parts of soybean seeds, which remain in the filter sack when pureed soybeans are filtered for the production of soymilk [179]. For each kilogram of soybean processed into soymilk, an equal weight or more of okara is produced [180]. Even though okara is frequently treated as industrial waste, it might be a good source of nutrients for human consumption, and studies have revealed that okara is a potential source of antioxidant components [181,182]. It might be useful as a weight-loss dietary supplement [183] and also protect the gut environment because of its antioxidant status and prebiotic effects [179,182]. Studies have also suggested that okara consumption could lead to a beneficial effect on plasma lipid levels [180].

A study evaluated the effect of inulin and okara flour on textural and sensory properties of probiotic soy yoghurt (SY) throughout 28 days of storage at 4 °C. Employing a 22 design, four formulations of SY produced from soymilk and fermented with an ABT-4culture (*Lactobacillusacidophilus* La-5, *Bifidobacterium animalis* Bb-12 and *Streptococcus thermophilus*) were studied: SY-C (control); SY-I (with inulin); SY-O (with okara); SY-IO (with inulin + okara).

The addition of okara and the refrigerated storage led to significant differences in the instrumental texture parameters of SY (*p* < 0.05). Inulin and okara did not affect SY sensory acceptability (*p* > 0.05), but there was a tendency for higher scores in the presence of inulin. On the other hand, the storage period, particularly at 21 days, was unfavorable regarding the acceptance of the different SY. The results showed that the addition of okara flour and the storage were significant factors to increase firmness of the soy yoghurts. SY acceptability was not affected by the incorporation of inulin or okara. These results suggest that okara, usually discarded as industrial waste, may be used in probiotic soy yoghurt, helping to increase the nutritional and functional properties without altering its acceptability [184].

## 5. Alternative Products from Agro-Industrial Waste

An innovative way of utilization could be related to the use of agricultural waste as alternative raw materials for obtaining building products, in particular, ceramic bricks.

The feasibility of using woody agricultural biomass wastes, such as grape and cherry seeds, and sawdust, as a pore forming agent; and sugar cane ash, as a silica precursor, in bricks, has been reported.

Sawdust and grape and cherry seeds, thanks to their organic substances content, during their combustion, bring an energetic support in the bricks’ firing phase and act as a pore forming agent. Usually, the addition of this kind of waste is limited to 10 wt. % in order to reach an equilibrium between positive (weight and shrinkage decrease and porosity increase) and negative (increase of water absorption and mechanical resistance decrease) effects. The results show that grape and cherry seeds, added at a percentage of 5 wt.% to a brick formulation, have better influence with respect to the sawdust, maintaining the mechanical properties of the fired brick (950 °C), showing modulus of rupture around 21–23 MPa with a weight reduction of 3%–10% (with respect to the standard one). Regarding the sugar cane ash, the addition of 5 wt. % improves the mechanical properties (modulus of rupture around 27 MPa) and no weight decrease is observed. These results confirmed the role played by this kind of agricultural waste, which thanks to its high silica content (61 wt. %) is capable of demonstrating a filler and plasticity reducing effect on the brick bodies [38].

Canete-Rodríguez et al. [185] focused on d-gluconic acid (GA), a common additive used in pharmaceutical, textile, building and, especially, food industries. GA is usually obtained through biological methods involving the partial oxidation of glucose. This acid provides an excellent example of how some production wastes and surpluses with high carbohydrate contents can be optimally exploited. This proposal is supported by summarizing the main properties, uses and production methods for GA and its derivatives (particularly its biotechnological derivatives). This review updates the content of previous reviews by authors such as Singh and Kumar [186], Anastassiadis and Morgunov [187], Ramachandran et al. [188], Rogers et al. [189], Roehr et al. [190] and Milsom and Meers [191]. The review reflects deeply on what are potentially the most useful aspects of this field with the hope of improving the exploitation of GA through the use of different types of surpluses and production wastes as raw materials and the development of new biotechnological production processes involving cellular microorganisms (fungi or bacteria) or their enzymes.

GA and its derivatives occur naturally in plants, fruits and other foods such as rice, honey, grapes, apples, meat, wine and vinegar [188]. Like many other organic acids, GA is involved in the metabolism of a number of living organisms. The acid and its derivatives have gained increasing interest in food, pharmaceutical, textile and building industries over the past 50 years. At present, the production of GA is estimated to amount to approximately 100,000 ton/year and to be almost exclusively biotechnological [192], with production costs ranging from 1.20 USD/kg (United States Dollar) for GA to 8.50 USD/kg for calcium gluconate and glucono-δ-lactone.

## 6. Disease Management with Nutraceuticals

Fruits, such as mango, banana, and those belonging to the citrus family, create a lot of waste residues in the form of peels, pulp, seeds, and stones. These residues represent a major disposal problem due to a lack of infrastructure to handle these large quantities of available biomass, a lack of processing facilities, and high processing costs, especially in developing countries. Such residues are of paramount importance due to the presence of phenolic compounds, which impart nutraceutical properties to fruit residues. The biological properties, such as anticarcinogenicity, antimutagenicity, antiallergenicity, and antiaging activity have been reported for both natural as well as synthetic antioxidants. Special attention is focused on the extraction of bioactive compounds from inexpensive or residual sources. Babbar et al. [193] characterized different phenolics present in the fruit residues, discussed the antioxidant potential of such residues and the assays used in determination of antioxidant properties. They also discussed various methods for efficient extraction of the bioactive compounds, and highlighted the importance of fruit residues as potential nutraceutical resources and biopreservatives.

Flavonoids present in citrus by-products have been extensively studied for antioxidative, anticancer, antiviral, and anti-inflammatory activities, effects on capillary fragility, and an observed inhibition of human platelet aggregation [194]. Recent research suggests that citrus fruits possess limonoids belonging to phytochemicals, which are highly oxygenated triterpenoids. Citrus limonoids (limonin, nomilin, and nomilinic acid) appear in large amounts in citrus juice and citrus tissues as water-soluble limonoid glucosides or in seeds as water-insoluble limonoid aglycones. Both seeds and leaves contain the limonoid azadirachtin [195].

Recent scientific studies have proved that the antioxidants are capable of protecting cells from free radical damage by inhibiting mutation and cancer because they have a scavenging role against reactive oxygen species, i.e., superoxide anion, hydroxyl radicals, and peroxy radicals [196]. Antioxidants act in various ways, which include forming complexes of redox-catalytic metal ions, scavenging of free radicals, and decomposition of peroxides. There is an association between individuals who have a diet rich in fresh fruits and vegetables and a decreased risk of certain forms of cancer and a longer life expectancy. Mantena et al. [197] studied the effect of grape seed proanthocyanidins in inducing apoptosis and inhibition of metastasis in both cultured breast and colon cancer cells. Pomegranate peel extracts have been shown to retard proliferation of cells in several different human cancer cell lines [198,199,200]. Pomegranate peel contains substantial amounts of polyphenols, such as ellagic acid and gallic acid [201]. The presence of these polyphenols in the pomegranate peel may be responsible for the antimutagenicity of peel extracts [202].

Several studies have shown that phenolic compounds reduce in vitro oxidation of Low Density Lipoproteins (LDL). Of them, phenolics with multiple hydroxyl groups are generally the most efficient for preventing lipid and LDL oxidation and therefore reduce the risk of atherogenesis [203,204]. An anthocyanin-rich extract from black rice decreased serum levels of triglycerides, total cholesterol and non-HDL cholesterol, and reduced the area of atherosclerotic plaques in apolipoprotein E-deficient mice [205].

Nutraceuticals have also been reported to have a protective effect against cardiovascular diseases (CVD). It has been found that increased levels of ROS and malondialdehyde (MDA), a metabolite that forms when ROS and oxidized Low Density Lipoproteins (LDL) attack fatty acids in cell membranes, result in cardiac arrest [206,207,208].

Tome-Carneiro et al. [209] focused on the time when the amount of polyphenols’ intake was measured, coming from randomized controlled trials (RCTs) of (poly) phenol-based supplements. They concluded that (poly) phenol-based nutraceuticals and functional foods might be indeed used as an adjunct therapy of CVD, but additional long-term RCTs with adequate numerosity and with clinically relevant end points are needed to provide unequivocal evidence of their clinical usefulness.

Nutraceuticals, such as curcumin, resveratrol, quericitin, and others have been long known for the cure and prevention of neurodegenerative disorders such Alzheimer’s disease (AD). AD is an age-related disorder and it sometimes gets coupled with metabolic disorders such as type 2 diabetes and obesity. However, the mechanism underlying this disease is still a subject matter of debate for the scientific community. On most of the occasions, its origin has been discovered in oxidative stress, decreased levels of plasma antioxidants, and total plasma antioxidant activity. Increased levels of reactive oxygen species (ROS) can result in significant damage to cell structures. This also leads to amyloid deposition and neurofibrillary tangles. Therefore, one of the early steps involved in AD pathogenesis is oxidative stress. Also, celluar mitrochondrial dysfunction resulting in the decreased production of ROS has been reported to exacerbate the disease [210,211].

Sahebkar et al. [212] provided an up-to-date review on the lipid-lowering effects of the most important nutraceuticals and functional foods. Based on current knowledge, nutraceuticals might exert significant lipid-lowering, and their use has several advantages: They have natural origins and are mainly extracted from natural products. They are mostly safe and very well tolerated. Their use is supported by the findings from randomized controlled trials and meta-analyses. The lipid-lowering effect of most nutraceuticals is multimechanistic, which makes them potential candidates for improving the effects of current lipid-lowering drugs when used in combination.

Questions that need to be addressed, include whether longer durations of therapy would result in a better response and the exact safety profile of nutraceuticals, especially at doses higher than those consumed in an average diet. Additionally, data regarding the effects of nutraceutical supplementation on the incidence of cardiovascular outcomes are lacking, and it is not clear whether the residual cardiovascular risk that remains after statin therapy can be modified by additional lipid lowering by nutraceuticals.

## 7. Nutraceutical Delivery Vehicles

There are two major approaches that can be used to improve the bioavailability of hydrophobic bioactives in foods. The first one is to isolate the bioactive components from their natural environment (such as a fruit or vegetable) and then to incorporate them into a suitable delivery system [213]. A wide range of colloidal delivery systems have been developed to encapsulate bioactive agents and have been reviewed in detail, such as molecular complexes, microemulsions, emulsions, liposomes, solid lipid nanoparticles, biopolymer particles, and microgels [214,215,216,217,218,219].

Lifestyle issues contribute to the development of obesity, type 2 diabetes, and cardiovascular disease. Together with appropriate diet and exercise, nutraceuticals may contribute to managing prevention at an early stage prior to therapeutic intervention. However, many useful food-derived bioactive compounds will not sufficiently permeate the small intestine to yield efficacy without appropriate oral delivery technology. The pharmaceutical industry uses commercialized approaches for oral delivery, including solubilizing technologies for small molecules, which could be applied to selected nutraceuticals with solubility issues. Systems currently being studied for labile and poorly permeable hydrophilic peptides and macromolecules include nanoparticles, intestinal permeation enhancers (PE) and mucolytics. These may also have potential for application to nutraceuticals with similar sub-optimal physicochemical characteristics.

Gleeson et al. [220] introduced factors, which effect oral delivery of four types of nutraceuticals, namely fatty acids, bioactive peptides, micronutrients, and phytochemicals. Factors preventing oral absorption can arise from molecule physicochemical characteristics, which influence solubility, stability, and epithelial permeability in the gastrointestinal tract (GIT). They highlighted the potential of selected delivery strategies to improve oral bioavailability of different types of nutraceuticals.

They found that there is an opportunity for the nutraceutical industry to leverage the pharmaceutical industry's progress in oral drug delivery. The use of delivery approaches using formulation with excipients or substances with a history of use in man has the potential to improve solubility, stability, or permeability of nutraceuticals, leading to improved oral bioavailability. Leveraging oral delivery formulation approaches across nutraceutical and pharmaceutical molecules will lead to synergies for both fields.

### 7.1. Liposomes

Liposomes are spherical microscopic lipid vesicles formed from phospholipids holding a small amount of solvent in which they exist. They serve as a delivery vehicle for both the hydrophilic and lipophilic compounds. When hydrophilic compounds are to be encapsulated, the aqueous center of liposome serves as a suitable pocket carrying the bioactive through the journey of the gastrointestinal (GI) tract digestion and absorption. Liposomes have been reported to enhance the oral bioavailability of a variety of nutraceuticals, such as curcumin and resveratrol, by entrapping them in the phospholipid bilayers [221,222]. The encapsulation results in improving the aqueous solubility, provides protection against unstable stimuli, modulating intestinal absorption, and facilitating lymphatic transport.

### 7.2. Phospholipid-Based

Phospholipids-based nanotherapeutics [223] are gaining popularity these days as delivery vehicles because they are biocompatible, biodegradable and possess properties that can enhance their bioavailability. When engaged in the aqueous environment, phospholipids simultaneously adapt themselves into a bilayer structure with polar heads facing outward and nonpolar tails pointing to the inner region of the bilayer structure.

### 7.3. Niosomes

Niosomes are composed mainly of hydrated nonionic surfactants in addition to cholesterol (CHOL) or its derivatives. The unique structures of niosomes make it capable of encapsulating both hydrophilic and lipophilic substances. This can be achieved by entrapping hydrophilic substances in the vesicular aqueous core or adsorbing them on the bilayer surfaces while the lipophilic substances are encapsulated by their partitioning into the lipophilic domain of the bilayers. Recently, niosomes have been used as delivery vehicles for the encapsulation of antioxidants as reported by Tavano et al. [224].

### 7.4. Emulsion-Based Delivery Vehicles

Because of their small particle size—usually >100 nm—and their ability to be formulated with generally recognized as safe (GRAS) material, emulsions are being used extensively for the delivery of nutraceuticals. The class includes microemulsions, nanoemulsions, and double emulsions. The nanoemulsion based oral formulations have been reported to significantly enhance the absorption of nutraceuticals such as curcumin, alpha tocopherol when compared to their nonencapsulated forms.

Emulsion based delivery systems that enhance the solubility of many other nutraceuticals such as Quericitin, curcumin, resveratrol, Vitamin D3, and so forth have also been reported in the literature [225,226].

### 7.5. Microemulsions/Nanoemulsions

Microemulsions (ME), with droplet diameters less than 100 nm, are spontaneous structures of water, oil, and surfactants. They offer the advantages of optical transparency, thermodynamic stability, long-term stability, and ease of preparation. Microemulsions have been employed over the past few years to improve the solubility of poorly water-soluble drugs/nutraceuticals in aqueous solutions either for the penetration into or absorption by cells in the pharmaceutical and functional foods. Phospholipids-based microemulsions (composed of food-grade ingredients, soybean oil and soybean lecithin) are being considered now as potential candidates for natural products because of their nanometer size, which enhances the trans membrane permeation as well as increasing the loading capacity for lipophilic nutraceuticals. It is believed that the small size allows passage through organ filtering and cellular membrane systems, both of which are highly desirable characteristics. A study conducted by researchers has established the superiority of soybean oil over ethyl oleate as the oil phase in curcumin microemulsion by the broadened microemulsion region in the phase diagram. The formulated microemulsion was found to have a cytotoxic effect on hepatocellular HepG2 cell lines [227].

On the other hand, nanoemulsions are assemblies similar to microemulsions. The only difference between them is that microemulsions are formed spontaneously and are thermodynamically stable whereas nanoemulsions require energy for their formulation and are kinetically stable systems. Wang et al. [228] successfully prepared high-speed and high-pressure homogenized O/W emulsions for delivery of curcumin using medium chain triacylglycerols (MCT) as oil and Tween 20 as an emulsifier with mean droplet sizes ranging from 618.6 nm to 79.5 nm.

Many highly hydrophobic bioactives, such as non-polar nutrients, nutraceuticals, and vitamins, have a relatively low or variable oral bioavailability. The poor bioavailability profile of these bioactives may be due to limited bioaccessibility, poor absorption, and/or chemical transformation within the gastrointestinal tract (GIT). The bioavailability of hydrophobic bioactives can be improved using specially designed oil-in-water emulsions consisting of lipid droplets dispersed within an aqueous phase. The bioactives may be isolated from their natural environment and then incorporated into the lipid phase of emulsion-based delivery systems. Alternatively, the bioactives may be left in their natural environment (e.g., fruits or vegetables), and then ingested with emulsion-based excipient systems. An excipient emulsion may have no inherent health benefits itself, but it boosts the biological activity of bioactive ingredients co-ingested with it by altering their bioaccessibility, absorption, and/or chemical transformation. McClements et al. [229] discussed the design and fabrication of excipient emulsions, and gave some examples that demonstrated their potential efficacy for improving the bioavailability of hydrophobic bioactives. The concept of excipient emulsions could be used to formulate emulsion-based food products (such as excipient sauces, dressings, dips, creams, or yogurts) specifically designed to increase the bioavailability of bioactive agents in natural foods, such as fruits and vegetables.

### 7.6. Ethosomes

Ethosomes are composed mainly of phospholipids (phosphatidyl choline, phosphatidyl serine, and phosphatitidic acid), ethanol (relatively high concentration, 40%–45%) and water. The higher content of ethanol makes ethosomes efficient permeation enhancers and they are usually added to vesicular systems to prepare elastic nanovesicles. The size of ethosomes varies between 30 nm to a few microns. However, ethosomes are low on stability and degrade once the ethanol evaporates. Apigenin (a plant flavanoid)- loaded ethosomes were prepared for local action at the skin. They showed higher skin deposition than liposomes or deformable liposomes both in vitro and in vivo. Ethosome-mediated apigenin delivery produced the strongest effect on ultra violet B (UVB)-induced skin inflammation by suppressing COX-2 levels [230].

### 7.7. Nanoencapsulation

Submicron emulsions, that is, solid lipid nanoparticles (SLNs), comprise a solid or semisolid lipid core structure and are considered an excellent controlled-release system capable of preventing burst release. They prolong the gastric retention time of bioactives. SLNs are a robust protective mechanism against the GI tract degradation activities, such as coenzyme Q10, retinol, citral, and peptides because of the reduced mobility of the lipid crystalline structure [231,232].

Moreover, there exists delivery protein-mediated nanocarrier systems for Hydrophobic Nutraceuticals (especially those derived from milk) which represent a well-tolerated system with few side effects [233].

## 8. Conclusions

Fruit and vegetable wastes (FVW) are produced in large quantities in markets and their recovery could be employed for the production of nutraceuticals and functional foods. In this chapter, we described the use of different plant and vegetable food residues as nutraceuticals and functional foods. Different nutraceuticals were explained. Their uses are well addressed along with their disease management and their action as nutraceutical delivery vehicles. Different vehicles have been described, such as liposomes, phospholipid-based, emulsion-based delivery vehicles, niosomes, microemulsions/nanoemulsions, ethosomes and nanoencapsulation.

## Figures and Tables

**Table 1:** Phenolic constituents as nutraceuticals from different fruit residues.

Fruit Phenolic	Waste Residue	Constituent	References
Apple	Peel and pomace	Epicatechin, catechins, hydroxycinnamates, phloretin glycosides, quercitin glycosides, procyanidins, chlorogenic acid, anthocyanins	[49,50,51,52,53,54,55,56,57,58,59,60,61,100,101,102]
Grapes	Seed and skin	Cinnamic acid, coumaric acid, caffeic acid, ferulic acid, chlorogenic acid, neochlorogenic acid, *p*-hydroxybenzoic acid, protocatechuic acid, vanillic acid, gallic acid, proanthocyanidins, quercetin 3-*O*-gluuronide, quercetin, resvaratrol, pullulan	[14,28,39,103,104]
Citrus	Peel	Hesperidin, naringin, eriocitrin, narirutin	[12,13,105]
Banana	Peel	Gallocatechin, anthocyanins, delphindin, cyaniding, catecholamine	[41,42,43,106,107,108]
Litchi	Pericarp, seeds	Cyanidin-3-glucosides, cyanidin-3-rutonoside, malvidin-3-glucoside, gallic acid, epicatechin-3-gallate	[69,70,71,72,73,74,109,110]
Mango	Kernel	Gallic acid, ellagic acid, gallates, gallotannins, condensed tannins	[111,112]
